# Supplementary material for: ERGA-BGE genome of the Spanish moon moth  Actias isabellae Graells, 1849: a nocturnal lepidopteran protected by the Habitats Directive
Source: Open Res Eur. 2025 Sep 16;5:175. Originally published 2025 Jun 30. [Version 2] doi: 10.12688/openreseurope.20658.2 (PMC12457886; doi:10.12688/openreseurope.20658.2)
Supplement: Peer review history

# Reviewer report: Reviewer response for version 2

**DOI:** 10.21956/openreseurope.23139.r60461

**Published:** 2025-09-23

**Authors:** Dimitar Dimitrov

**Affiliations:** 1 University of Bergen, Bergen, Norway

I think that the authors have addressed satisfactory my comments on the previous version of the manuscript.

Are sufficient details of methods and materials provided to allow replication by others?

Yes

Is the rationale for creating the dataset(s) clearly described?

Yes

Are the datasets clearly presented in a useable and accessible format?

Yes

Are the protocols appropriate and is the work technically sound?

Yes

Reviewer Expertise:

molecular systematics, phylogenetics, phylogenomics, macroecology, macroevolution

I confirm that I have read this submission and believe that I have an appropriate level of expertise to confirm that it is of an acceptable scientific standard.

# Reviewer report: Reviewer response for version 1

**DOI:** 10.21956/openreseurope.22348.r56370

**Published:** 2025-08-15

**Authors:** Dimitar Dimitrov

**Affiliations:** 1 University of Bergen, Bergen, Norway

In the present article, Vila et al present a reference genome assembly of the Spanish moon moth. This species has been included as protected in the Bern convention and the EU habitat directive, hence it is of interest for conservation biologists. In that respect generating a high-quality reference genome can help future population genomics work. The present genome also adds to our knowledge of lepidopteran genomes and improves taxon samples of potential comparative genomics studies focused on Lepidoptera.

 The present work is part of the ERGA effort to compile a reference genomes atlas of European species, and the authors follow the protocols established by the ERGA consortium. In that respect, protocols are well established and information about sequencing and bioinformatics pipelines is available. Overall, the article is concise and contains all necessary information. However, I think that in few places it could benefit from additional work to improve the flow and style. In that respect here are few suggestions to the authors:

 Abstract – I would suggest that the authors consider reworking a bit the abstract. For example, they can start with a general introductory sentence, then present their findings and finally speculate about the broader impact.

 Key words – some of the key words are already present in the title.

 “Recent phylogenomic analyses have supported a change in the scientific name of the Spanish moon moth, ..” that is odd wording. The analyses have supported a transfer to other genus, hence the change of name.

 “The estimated genome size, based on ancestral taxa, is “ – that is strange wording. Did you have direct information about ancestral taxa genome size? I guess you mean, that information comes from ancestral state reconstruction analyses … or similar.

 “The nuclear microsatellite markers used in that work ( Vila *et al.*, 2010) also revealed a west–east cline in allele frequencies along the Central Pyrenees that causes low overall genetic differentiation within and around the Ordesa y Monte Perdido National Park ( González-Castellano *et al.*, 2023).” – reading this sentence I get the impression that “that work” refers to Marí-Mena *et al.* (2016) mentioned in the previous sentence. However, here Ville et al., 2010 is cited which is confusing. Further, still talking about different aspects of “that work” two other different papers are cited. Maybe all these works are relevant and thus it is probably better to add few sentences here to make that clear and avoid confusion.

 Can you explain a bit why BioSample SAMEA116288378 was used as reference.

Are sufficient details of methods and materials provided to allow replication by others?

Yes

Is the rationale for creating the dataset(s) clearly described?

Yes

Are the datasets clearly presented in a useable and accessible format?

Yes

Are the protocols appropriate and is the work technically sound?

Yes

Reviewer Expertise:

molecular systematics, phylogenetics, phylogenomics, macroecology, macroevolution

I confirm that I have read this submission and believe that I have an appropriate level of expertise to confirm that it is of an acceptable scientific standard, however I have significant reservations, as outlined above.

# Response

**Published:** 2025-09-09

**Authors:** Chiara Bortoluzzi

**Affiliations:** Swiss Institute of Bioinformatics, Lausanne, Vaud, Switzerland

The abstract was changed as suggested. We have changed the wording to: “High-quality reference genomes are critical resources for understanding biodiversity and supporting conservation efforts. We present the chromosome-level assembly of the Spanish moon moth, Actias isabellae (Graells, 1849), a nocturnal lepidopteran protected under the EU Habitats Directive. The assembly spans 0.56 Gb across 31 pseudomolecules, including the Z chromosome, with a contig N50 of 18.9 Mb and a scaffold N50 of 20.4 Mb. The mitochondrial genome was also assembled into a 15,247 bp circular sequence. Annotation identified 11,805 protein-coding genes and 2,238 non-coding genes, with a BUSCO completeness of over 94%. Notably, no W chromosome was detected, suggesting a ZZ/Z0 sex determination system. This reference genome provides an essential foundation for studying sex chromosome evolution in Lepidoptera and enables advanced population genomics monitoring of this protected species. More broadly, it contributes to ongoing efforts within the European Reference Genome Atlas and the Earth BioGenome Project to harness genomics for biodiversity conservation”.   Keywords were changed as suggested. These are the key words in the revised version: “Graellsia isabellae, Biodiversity Genomics Europe, Insecta, Lepidoptera, Saturniidae”.   We agree that the wording regarding the recent phylogenetic analyses was misleading. Accordingly, we have changed the text to: “Recent phylogenomic analyses have supported the transfer of the Spanish moth from the genus Graellsia (Graells, 1849) to genus Actias (Graells, 1849).”   The reviewer is correct that we do not have information about ancestral taxa - this was a mistake. We have changed the wording to: “The estimated genome size, estimated by Genomes on a Tree (GoaT) by ancestral state reconstruction, is”. The estimated genome size reported in the main text is the one reported by GoaT ( https://goat.genomehubs.org/), which is used prior to sequencing to help calculate sequencing effort (e.g. library prep reagents and number of Revio flow cells). We are aware that the value reported in GoaT might not be exact, but it is an educated guess especially for taxa and lineages with no genome size available. The assumption that a species might have its genome size within the range described for its taxonomic group is the basis for GoaT estimates: the median of genome sizes are computed from taxa with direct measurements and propagated to the closest common ancestor, then back to fill the missing data. More information on how GoaT calculates ancestral values to propagate can be found in https://wellcomeopenresearch.org/articles/8-24/v1.    We modified the text accordingly to state this more clearly, specifying the source of the markers (Vila et al. 2010) used for the 2016 and 2023 studies.

   The specimen used for the assembly is the one with BioSample SAMEA114400479. This specimen is a female and was selected because it is the heterogametic sex. However our analyses revealed an X0 determination system and therefore  is ‘hemizygous’ for the sex chromosome. We changed the main text accordingly to clarify this.

# Reviewer report: Reviewer response for version 1

**DOI:** 10.21956/openreseurope.22348.r56364

**Published:** 2025-08-15

**Authors:** Jiufeng Wei

**Affiliations:** 1 Shanxi Agricultural University, Jinzhong, Shanxi, China

The manuscript, titled "ERGA-BGE genome of the Spanish moon moth Actias isabellae Graells, 1849: a nocturnal lepidopteran protected by the Habitats Directive," reports the sequencing and assembly of a lepidopteran species. The chromosome-level genome assembly spans approximately 0.56 Gb in length. Both the sequencing methods and analyses were adequate. The results are reliable, and I recommend to index the manuscript.

Are sufficient details of methods and materials provided to allow replication by others?

Yes

Is the rationale for creating the dataset(s) clearly described?

Yes

Are the datasets clearly presented in a useable and accessible format?

Yes

Are the protocols appropriate and is the work technically sound?

Yes

Reviewer Expertise:

Phylogenomic analysis of Insect.

I confirm that I have read this submission and believe that I have an appropriate level of expertise to confirm that it is of an acceptable scientific standard.

# Response

**Published:** 2025-09-09

**Authors:** Chiara Bortoluzzi

**Affiliations:** Swiss Institute of Bioinformatics, Lausanne, Vaud, Switzerland

We thank the reviewer for their positive response and for approving our Genome Report.
